# Development of a social activities scale for community‐dwelling older people requiring support

**DOI:** 10.1002/nop2.578

**Published:** 2020-07-26

**Authors:** Michiyo Hirano, Kazuko Saeki, Izumi Ueda

**Affiliations:** ^1^ Faculty of Health Sciences Hokkaido University Hokkaido Japan; ^2^ Hokkaido University Hokkaido Japan; ^3^ School of Health Sciences Sapporo Medical University Hokkaido Japan

**Keywords:** aged, instrument construction, older people requiring support, social participation

## Abstract

**Aim:**

This study aims to develop the Social Activities Scale for Community‐Dwelling Older People Requiring Support (SASOS).

**Design:**

This study is a cross‐sectional investigation.

**Methods:**

The participants were healthy older people (HOP; *N* = 140) and those requiring support (OPRS; *N* = 250). An anonymous questionnaire included items on SASOS, subjective health and ikigai (Japanese well‐being concept). Criterion‐related validity was examined using the Social Activity Index for Elderly People (SAI‐E).

**Results:**

Seventy‐five HOP and 157 OPRS provided effective responses. The scale (α = 0.805) had three subscales: "interactions with friends and neighbors (F1),” "close relationships with family (F2)” and "interactions with others through activity programs (F3).” SASOS and SAI‐E scores were correlated (*r* = .558, *p* < .01), indicating criterion‐related validity. In known‐groups validity analysis, F1 were significantly higher among HOP and F3 were significantly higher among OPRS. Total scores correlated with perceived health (*r* = .240, *p* < .01) and ikigai (*r* = .419, *p* < .01).

## 1 INTRODUCTION

Japan's population is ageing rapidly. In 2017, older people (age > 65 years) accounted for 17.4% of the population in 36 OECD member countries; the proportion was largest in Japan (27.7%) (OECD, 2019). This proportion in OECD countries is expected to reach 27.1% in 2050 (OECD, 2017). Thus, Japan must implement preventive care ahead of the rest of the world.

Social activities improve cognitive ability (Choi, Park, Cho, Chun, & Park, 2016), motor function (Buchman, Boyle, & Wilson, 2009) and subjective health status (Okamoto, Okada, & Shirasawa, 2005) and reduce mortality (Steinbach, 1992), among older people. They are related to reduced risks of incident disorders in daily activities, exercise and instrumental activities of daily living (IADL) (James, Boyle, Buchman, & Bennett, 2011). They improve well‐being (James, Boyle, et al., 2011) and purpose in life [Japanese concept; *ikigai*] (Okamot, 2010). Social activities, especially with friends, are associated positively with life satisfaction (Lemon, Bengtson, & Peterson, 1972) and negatively with depression (Lee and Kim, 2014). Spending time with friends offers a survival advantage over other leisure activities (Maier & Klumb, 2005). Thus, social activities are beneficial for older people, but they have been shown to have no effect among older people with reduced IADL ability requiring support.

Lemon et al. (1972) categorized social activities as formal, informal and solitary. Formal and informal activities include attending church/religious services, senior clubs/centres and school/family reunions; face‐to‐face interaction with close friends and children; and communication with children by telephone or letters (Lee and Kim, 2014). Formal social activities of older people also include participation in organized/political groups (Choi, Park, Cho, Chun, & Park, 2016; Glei et al., 2005), volunteer activities (Choi et al., 2016; Glei et al., 2005; James, Wilson, Barnes, & Bennett, 2011), religious organizations(Choi et al., 2016; Glei et al., 2005; James, Wilson, et al., 2011), leisure/culture/sports clubs (Choi et al., 2016), games (Glei et al., 2005; James, Wilson, et al., 2011), sports events and off‐track betting (James, Wilson, et al., 2011), travel (James, Wilson, et al., 2011) and interaction with friends/neighbours(Glei et al., 2005; James, Wilson, et al., 2011).

Hirano et al. developed gender‐specific social activities scales based on the characteristics of social activities of older people requiring support. For men, these activities were daily interactions with familiar people, maintaining close relationships with family and interactions with others through activity programs (Hirano, Saeki, & Ueda, 2018). For women, they were interactions with familiar people, consulting care service providers and performing proactive creative activities at home (Hirano, Kawahara, & Saeki, 2015). Because of reduced IADL ability, the range and content of activities of older people requiring support differ from those of healthy older people. Thus, a social activity scale must be developed for older people requiring support. Currently available social activity scales are gender specific (Hirano et al., 2018, Hirano, Kawahara, & Saeki, 2014); an easy‐to‐use scale for both genders is lacking.

### 1.1 Purpose of the research

In this study, the Social Activities Scale for Community‐Dwelling Older People Requiring Support (SASOS) was developed, with consideration of social activity characteristics by gender. This scale enables the collection and analysis of basic data informing the implementation of interventions targeting the social aspects of older people requiring support and improvement of their long‐term preventive care and health.

## 2 METHODS

### 2.1 Term definition

Based on previous studies (Hirano et al., 2015, Hirano et al., 2018; Lemon et al., 1972), social activities of older people requiring support were defined in this study as "interactions with family and friends and participation in groups/organizations."

### 2.2 Conceptual framework

Construct validity was examined based on the operational term and previous findings (Hirano et al., 2014,2018). The SASOS assesses three types of social activity: interactions with friends and neighbours, close relationships with family and interactions with others through activity programs. Because subjective views of health and *ikigai* are related to social activities of older people, they were used to examine the convergent validity of the SASOS. Criterion‐related validity was examined using the Social Activity Index for Elderly People (SAI‐E) (Hashimoto et al., 1997), which has confirmed reliability and validity, as an external criterion.

### 2.3 Preliminary study

We developed gender‐specific social activity scales. The 10‐item scale for men (Hirano et al., 2018) has three subscales: daily interactions with familiar people, close relationships with family and interactions with others through activity programs. The 15‐item scale for women (Hirano et al., 2014, 2015) has three subscales: interactions with familiar people; performing proactive, creative meal‐related activities; and consulting care service providers. Based on these scales and qualitative findings (Hirano et al., 2014,2015,2011,2018,2017), we drafted a three‐concept, 17‐item SASOS covering interactions with friends and neighbours (8 items), close relationships with family (4 items) and interactions with others through activity programs (5 items; Table 1). Extracted items addressed social activities that were (a) common to men and women (nos. 3, 7, 9, 10), (b) on only one scale but potentially undertaken by both genders (men: nos. 1, 2, 6, 11, 13, 14; women: nos. 5, 12, 15–17) and (c) previously found to be valued by older people requiring support (nos. 4, 8) (Hirano, Saeki, & Kawahara, 2011; Hirano, Saeki, Ueda, Honda, & Mizuno, 2017). These items enable the assessment of mutual interactions with familiar people in familiar places among older people requiring support with limited activity ranges due to poor IADL ability.

**Table 1:** Item analysis of SASOS. *N* = 157

	Items	Mean	*SD*	G‐P analysis	I‐T correlation analyses, correlation coefficient
**"Interactions with friends and neighbors"**					
1	Maintaining a close relationship with neighbours	2.81	1.30	***, a	0.54
2	Talking with the people in neighbourhood, checking on each other's condition	2.80	1.24	***, a	0.47
3	Communicating with friends by letter or telephone to check on each other's condition	2.92	1.20	***, a	0.44
4	As much as possible, helping friends in trouble	2.15	1.12	***, a	0.50
5	Serving friends tea and cakes	2.56	1.11	***, a	0.38
6	Amicably greeting neighbours	3.24	1.14	***, a	0.46
7	Enjoying a pleasant time with a close friend	3.35	1.03	***, a	0.46
8	Seeing and talking with former colleagues	1.81	1.04	***, a	0.35
**"Close relationships with family"**					
9	Enjoying meals and chatting with family	3.37	1.38	***, a	0.41
10	Along with family members living together or apart, having a relaxing time	3.34	1.39	***, a	0.40
11	Having family and relatives help with daily activities	3.22	1.43	***, a	0.27
12	Serving family tea and cakes	3.08	1.35	***, a	0.44
**"Interactions with others through activity programs"**					
13	Observing activities and state of others at a senior club, hobby meeting or day service centre	3.27	1.24	***, a	0.37
14	Enjoying conversation with others at a senior club, hobby meeting or day service	3.42	1.12	***, a	0.36
15	Consulting a long‐term care service provider (e.g. staff member, helper at a comprehensive community support centre or day service) about concerns regarding health and physical condition	3.09	1.06	***, a	0.48
16	Getting advice from a long‐term care service provider (e.g. staff member, helper at a comprehensive community support centre or day service) about medical treatment	2.85	1.10	***, a	0.38
17	Consulting a long‐term care service provider (e.g. staff member, helper at a comprehensive community support centre or day service) about illness and physical condition	2.90	1.17	***, a	0.36

*** *p* < .001, G‐P analysis used *t* test. I‐T correlation analyses used Spearman's rank correlation coefficient.

### 2.4 Main study

#### 2.4.1 Long‐term care insurance in Japan

In Japan, people aged >65 years are eligible for long‐term care certification. Standardized needs assessment for this certification comprises seven levels: support levels 1 and 2 (requiring support for daily activities) and care levels 1–5 (requirement for continuous care). Support and care services are similar, with the critical difference that support services offer preventive long‐term care to "[maintain] or [enhance] the ability to become independent" (Tsutsui & Muramatsu, 2007) Public health nurses, certified social workers and senior care managers at comprehensive community support centres provide such preventive care management (Tsutsui & Muramatsu, 2007).

### 2.5 Design and sample

#### 2.5.1 Study area

This study was performed in three municipalities in Prefecture A, northern Japan, that provided research consent. Prefecture A has a population of 5.3 million, including 1.6 million people aged >65 years and 98,000 people with support certification (Report on project of long‐term care, 2015).

#### 2.5.2 Participants

Scale development was performed with a convenience sample of 250 older people requiring support without dementia and 140 healthy older people residing in the three municipalities. The factorial validity of the SASOS was examined with older people requiring support and healthy older people served as a known group in the examination of known‐groups validity.

In estimating the required sample size, the following parameters were used to ensure the statistical power of testing differences in SASOS scores between healthy older people and older people requiring support: significance level = 5%, expected between‐group difference = 1.6, standard deviation = 4.4 and statistical power = 80%. The expected between‐group difference and *SD* were calculated based on previous results (Hirano, 2014). Calculations determined that each group should contain ≥ 120 people.

Comprehensive community support centre administrators in the three municipalities were asked to help recruit older people requiring support and referred 250 patients. Municipal social welfare councils were asked to help recruit healthy older people and referred 140 patients participating in activity programs at elder welfare centres.

#### 2.5.3 Data collection

Between September 2017–February 2018, centre care staff conducted individual interviews using an anonymous questionnaire at the homes of older people requiring support. Social welfare council members conducted the same survey among healthy older people.

#### 2.5.4 Measures

The survey comprised the SASOS, SAI‐E and items pertaining to respondents’ characteristics, subjective health status (on a 4‐point scale) and *ikigai* (Imai, Osada, & Nichimura, 2012). SASOS responses are structured by a 5‐point Likert scale representing social activity frequency (1 = never and 5 = almost every day). The SAI‐E consists of 21 items in 4 domains (individual activities, social participation/volunteer activities, learning activities and work). Responses in the first three domains are coded as 1 (always/sometimes) and 0 (never) and those in the work domain are dichotomized as 1 (yes) and 0 (no). *Ikigai*, a Japanese well‐being concept defined operationally as "consciousness consisting of optimistic/positive views of the current life, active/positive attitude towards the future and self‐identity in the society," (Imai, Osada, & Nichimura, 2009) was measured using the 9‐item, 3‐domain Ikigai scale, with responses structured by a 5‐point Likert scale (1 = strongly disagree and 5 = strongly agree); higher scores reflect greater *ikigai*.

#### 2.5.5 Analytic strategy

Items were selected by correlation analysis, good–poor analysis and item–total correlation analysis. Scale reliability was examined by assessing internal consistency using Cronbach's α. The construct validity of the SASOS was examined based on operational term definitions and previous findings (Hirano et al., 2014,2011,2018,2017). Criterion‐related validity was examined using the SAI‐E, which has confirmed reliability and validity, as an external criterion in Spearman's rank correlation analysis. Factorial validity was examined by exploratory and confirmatory factor analyses using data from older people requiring support. For exploratory factor analysis (including the Kaiser–Meyer–Olkin test of sampling adequacy and Bartlett's test of sphericity), the factor number was set to 3 based on the conceptual framework and scree plot criteria and promax rotation was performed. Items with factor loadings ≥ 0.4 were selected. For confirmatory factor analysis, the maximum likelihood method and goodness‐of‐fit indices [goodness of fit index (GFI), adjusted goodness of fit index (AGFI), confirmatory fit index (CFI) and root mean square error of approximation (RMSEA)] were used. To assess known‐groups validity, SASOS scores were compared between healthy older people and older people requiring support using the Mann–Whitney *U* test. Convergent validity was examined by correlating SASOS scores from older people requiring support with perceived health and *ikigai* scores. Statistical analyses were performed with IBM SPSS 22.0 and IBM SPSS Amos 23.0, with a significance level of 0.05.

### 2.6 Ethical considerations

This study was conducted after obtaining approval from the ethics committee of the researchers’ affiliated university (approval no. 17–74, 20 September 2017). Centre directors and participants were given written explanation of the study's purpose and content and the ability to withdraw voluntarily and provided oral consent at the beginning of the survey.

## 3 RESULTS

Responses were received from 190 (64.0%) older people requiring support and 85 (60.7%) healthy older people; data from 157 older people requiring support (62.8% effective response rate) and 75 healthy older people (53.6% effective response rate) who responded to all SASOS items were included in the analysis.

### 3.1 Participant characteristics

The mean age of older people requiring support [*N* = 127 (80.9%) women] was 82.7 (*SD* 6.5) years, and 92 (58.6%) older people requiring support lived alone. The mean age of healthy older people [*N* = 50 (66.7%) women] was 74.6 (*SD* 5.7)years, with 31 (42.5%) people living with spouses.

Most older people requiring support perceived that they were "fairly healthy" [*N* = 89 (56.7%)] or "not so healthy" [*N* = 47 (29.9%)]; 143 (91.1%) older people requiring support received preventive long‐term care, most frequently day services [*N* = 89 (56.7%)]. Most healthy older people perceived that they were "fairly healthy" [*N* = 53 (70.7%)] or "not so healthy" [*N* = 10 (13.3%); Table 2].

**Table 2:** Demographic Characteristics of Survey Respondents.* N* = 232

Characteristic	Older people requiring support (*N* = 157)		Healthy older people (*N* = 75)	
	*N*	%	*N*	%
Sex				
Men	30	19.1%	24	32.0%
Women	127	80.9%	50	66.7%
Missing	–	–	1	1.3%
Age (years)				
65～74	19	12.1%	38	50.7%
75+	137	87.3%	35	46.7%
Missing	1	0.6%	2	2.7%
Mean ± *SD*	82.7 ± 6.5		74.6 ± 5.7	
Living arrangements				
With others	26	16.6%	19	26.0%
With spouse	34	21.7%	31	42.5%
Alone	92	58.6%	23	31.5%
Other	5	3.2%	–	–
Subjective health status				
Very healthy	7	4.5%	10	13.3%
Fairly healthy	89	56.7%	53	70.7%
Not so healthy	47	29.9%	10	13.3%
Not healthy	14	8.9%	1	1.3%
Missing	‐	‐	1	1.3%
Certification of long‐term care need				
Support Level 1	80	55.9%		
Support Level 2	63	44.1%		
Use of care services				
Yes	143	91.1%		
No	11	7.0%		
Missing	3	1.9%		
Preventive long‐term care^***^, ^a^				
Day service	89	56.7%		
Home care	52	33.1%		
Outpatient rehabilitation	22	14.0%		
Visiting nursing	3	1.9%		
	Mean ± *SD*	range		
SAI‐E				
Individual activities^b^, ^*^	4.61 ± 2.34	(0–10)		
Social participation/ volunteer activities^c^, ^**^	1.37 ± 1.64	(0–6)		
Learning activities^c^, ^**^	0.28 ± 0.61	(0–4)		
Work^c^, ^**^	0.03 ± 0.18	(0–1)		
Total score^b^, ^*^	6.30 ± 3.85	(0–21)		
Ikigai				
Consciousness consisting of Optimistic/positive views of the current life^d^	10.57 ± 2.78	(3–15)		
Active/positive attitude towards the future^d^	9.01 ± 3.25	(3–15)		
Self‐identity in the society^b^, ^*^	8.85 ± 3.19	(3–15)		
Total score^b^, ^*^	28.4 ± 7.71	(9–45)		

Note.

Home care includes housework and personal care, but excludes the provision of nursing and medical care. Day service is adult day care with pick‐up and drop‐off services, in which everyday care and training are provided during the day at retirement homes or day‐care centres.

^a^ Multiple answers;

^b^ *N* = 154;

^c^ *N* = 155;

^d^ *N* = 156.

### 3.2 SASOS item selection

"Getting advice from a long‐term care service provider (e.g., staff member, helper at a comprehensive community support center or day service) about medical treatment" and "consulting a long‐term care service provider (e.g., staff member, helper at a comprehensive community support center or day service) about illness and physical condition" correlated strongly (*r* ≥ .8) with the SASOS. Mean item scores were 1.81–3.42 (*SD* 1.03–1.43), with only "seeing and talking with former colleagues" showing a floor effect. In the good–poor analysis, the group with higher scores had significantly higher scores on all items (*p* < .001). In the item–total correlation analysis, all items showed significant positive correlations (*r* ≥ .2). Based on these results, "consulting a long‐term care service provider about illness and physical condition" and "seeing and talking with former colleagues" were excluded from subsequent analyses (Table 1).

### 3.3 SASOS properties

#### 3.3.1 Factorial validity and internal consistency

Kaiser–Meyer–Olkin and Bartlett's tests of the 15 SASOS items yielded values of 0.754 and 1,089.918 (*p < *.001), respectively, confirming the adequacy of the factor analysis. Three factors were identified: interactions with friends and neighbours (F1), close relationships with family (F2) and interactions with others through activity programs (F3; Table 3).

**Table 3:** Factor Pattern Matrix of the Social Activity Scale for Community‐Dwelling Older People Requiring Support (Principal Factor Method, Promax Rotation) (*N* = 157, α = 0.805)

Factor Item and Cronbach's Alpha Coefficient	Factor loading			Communa‐lity
	Factor 1	Factor 2	Factor 3	
Factor 1. "Interactions with friends and neighbors" (α = 0.842)				
Maintaining a close relationship with neighbours	**0.783**	0.056	−0.049	0.614
Talking with the people in neighbourhood, checking on each other's condition	**0.728**	0.042	−0.076	0.521
Communicating with friends by letter or telephone to check on each other's condition	**0.668**	−0.058	0.116	0.432
As much as possible, helping friends in trouble	**0.657**	−0.092	0.038	0.485
Serving friends tea and cakes	**0.627**	−0.098	0.007	0.384
Amicably greeting neighbours	**0.616**	0.114	−0.061	0.400
Enjoying a pleasant time with a close friend	**0.489**	0.005	0.189	0.320
Factor 2 "Close relationships with family" (α = 0.852)				
Enjoying meals and chatting with family	−0.060	**0.972**	0.020	0.933
Along with family members living together or apart, having a relaxing time	−0.073	**0.819**	0.117	0.689
Having family and relatives help with daily activities	−0.058	**0.700**	−0.033	0.476
Serving family tea and cakes	0.257	**0.592**	−0.085	0.451
Factor 3. "Interactions with others through activity programs" (α = 0.771)				
Observing activities and state of others at a senior club, hobby meeting or day service centre	−0.028	−0.029	**0.856**	0.717
Enjoying conversation with others at a senior club, hobby meeting or day service	−0.023	−0.025	**0.796**	0.622
Consulting a long‐term care service provider (e.g. staff member, helper at a comprehensive community support centre or day service) about concerns regarding health and physical condition	0.100	0.079	**0.532**	0.338
Getting advice from a long‐term care service provider (e.g. staff member, helper at a comprehensive community support centre or day service) about medical treatment	0.041	0.035	**0.488**	0.256
Factor contribution	3.336	2.625	2.227	
Factor Correlations F1		0.165	0.238	
F2			0.121	

**Figure 1:**
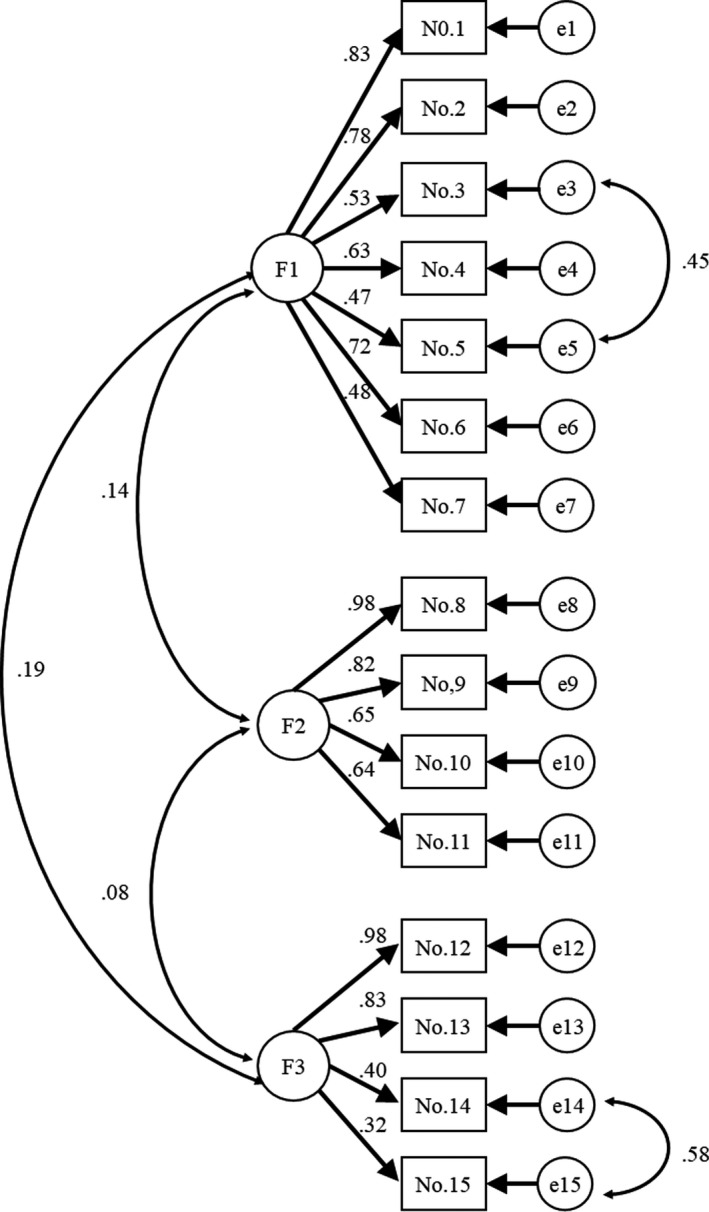
Social activity scale for community‐dwelling older people requiring support: confirmatory factor analysis

Maximum likelihood estimation indicated acceptable adjustment by the three‐factor oblique model. Goodness of fit results were as follows: GFI = 0.888, AGFI = 0.842, CFI = 0.941 and RMSEA = 0.068. Standardized coefficients for the three factors (i.e. latent variables) for each observed variable were 0.322–0.979 (*p* < .001), and all estimates were significant (*p* < .05) (Figure 1). The numbers above factor loadading 0.4 are shown in bold. Cronbach's α values for the 15‐item SASOS and F1–F3 were 0.805, 0.842, 0.852 and 0.771, respectively (Table 3).

#### 3.3.2 Criterion‐related, known‐groups and convergent validity

The coefficient of correlation between the SASOS and SAI‐E total scores was 0.558 (*p* < .01). The SAI‐E “work” and SASOS F2 items did not show correlations ≥ 0.2 (Table 4).

**Table 4:** Correlations between the SASOS and SAI‐E, Subjective health status and Ikigai. *N* = 155

SAI‐E	SASOS							
	Factor 1		Factor 2		Factor 3		Total score of the SASOS	
Individual activities	0.606	c, **	0.170	b, *	0.239	c, **	0.528	c, **
Social participation/volunteer activities	0.431	c, **	0.078		0.244	c, **	0.411	c, **
Learning activities	0.324	c, **	0.121		0.022		0.262	c, **
work	0.082		−0.009		0.033		0.067	
Total score of the SAI‐E	0.617	c, **	0.186	b, *	0.253	c, **	0.558	c, **
Subjective health status	0.216	b, *	<0.001		0.203	b, *	0.240	c, **
Ikigai (*N* = 154)	0.379	c, **	0.235	c, **	0.217	b, *	0.419	c, **

* *p* < .05,

** *p* < .01, Spearman's rank correlation coefficient.

Possible SASOS score ranges are 5–35 (7 items) for F1 and 4–20 (4 items each) for F2 and F3. Among older people requiring support, mean total and F1–F3 scores were 45.5 (*SD* 9.5), 19.8 (*SD* 5.9), 13.0 (*SD* 4.6) and 12.6 (*SD* 3.5), respectively; among healthy older people, they were 43.5 (*SD* 10.2), 21.6 (*SD* 6.1), 12.6 (*SD* 4.5) and 9.3 (*SD* 3.1), respectively. F1 scores were significantly higher (*p* = .023) and F3 scores were significantly lower (*p* < .001) among older people requiring support than among healthy older people (Mann–Whitney *U* test). No significant difference was observed in F2 or SASOS total scores.

Total SASOS scores correlated with respondents’ perceived health (*r* = .240, *p* < .01) and *ikigai* (*r* = .419, *p* < .01). F1 and F3 scores correlated with perceived health and *ikigai* and the F2 score correlated with *ikigai* (all *r* ≥ .2; Table 4).

## 4 DISCUSSION

### 4.1 SASOS reliability and validity

The SASOS showed adequate internal consistency (*α* > 0.7) (Bland & Altman, 1997). The three expected concepts were identified as factors, reflecting adequate construct validity. The GFI was slightly lower than the recommended value (0.9) and the RMSEA was slightly higher than recommended (≤0.05), likely reflecting the small sample, as the GFI increases with sample size (Gerbing & Anderson, 1992) and RMSEA ≤ 0.08 is a reasonable error estimate (Browne & Cudeck, 1992). The results show that the SASOS contains items that do not contribute to the goodness of fit, but given the acceptability of its fit indices, it meets certain criteria.

The F2 and total SASOS scores did not differ between healthy older people and older people requiring support. F1 scores were significantly higher among healthy older people, and F3 scores were significantly higher among older people requiring support. These results can be interpreted from the perspective of the hierarchical‐compensatory model (Cantor, 1979), which is based on the concept that older people seek support hierarchically. When high‐priority people are lacking and/or support cannot be provided, the next‐ranking people are substituted and complement support provision (Cantor, 1979). Replacing support in this model with social activity, we can infer that older people requiring support use F3 programs as complementary social activity resources, given that they have less F1 interaction than do healthy older people due to decreased IADL. Such compensation explains the lack of a significant difference between groups in the total SASOS score. The lack of difference in the F2 score, despite differences in living arrangements, between older people requiring support and healthy older people can be attributed to the inclusion of co‐resident and separately residing family members in F2 items. Saito (2008) found that the frequency of interaction with children living separately and/or friends tended to increase with a decreasing number of co‐resident people.

Respondents’ perceived health correlated weakly with the total SASOS score and not with the F2 score, possibly because 39% of patients were older people requiring support with poor perceived health; their social activities may be related instead to *ikigai*, which correlated with all SASOS scores. The results suggest that social activities contribute to the *ikigai* of older people requiring support, although no causal relationship was established. They also suggest that F2 interactions are viable social activities for older people requiring support, regardless of health status.

The moderate correlation between SASOS and SAI‐E scores shows that the SASOS is a measure of older people's social activities. The SAI‐E "work" score was not correlated with SASOS factor scores, indicating that work cannot be considered a social activity of older people requiring support, as in previous studies (Hirano et al., 2018, 2015). F1 and F3 scores correlated with the SAI‐E "individual activities" and "social participation" scores, and the F1 score correlated moderately with the "learning activities" score. F1 activities are similar to those of healthy older people, whereas F3 activities are specific to older people requiring support and thus may contain elements of healthy older people's "individual activities" and "social participation." In F3, "consulting a long‐term care service provider about concerns regarding health and physical condition" seems to be an additional social activity for older people requiring support. F2 scores correlated weakly only with SAI‐E "individual activity" scores. In previous studies, social activities have been treated mainly as formal. The SASOS covers informal and formal activities, reflecting the actual social activities of older people requiring support and thus may be a useful and effective scale.

### 4.2 Clinical application

This study showed that social activities are associated with the *ikigai* of older people requiring support. The promotion of social activities using SASOS concepts may help to develop *ikigai* in older people requiring support. F1 and F3 are particular targets, as they may contribute to the maintenance and increase the range of social activities and increase life satisfaction and physical function.

SASOS use can contribute to individual care, by enabling care management (with incorporation of social activities) and assessment of care effects. In addition, SASOS data from older people requiring support can be used to clarify the actual situations and characteristics of regional social resources for community development.

### 4.3 Limitations

This study has three limitations. First, goodness‐of‐fit results, The GFI and AGFI was slightly than recommended. Additionally, generalization of the study results is difficult because the data were from a small group of older people requiring support, which included fewer men than women. The validity of the scale for men and women should be verified further in larger samples of older people requiring support. Second, because this study was cross‐sectional and used an anonymous questionnaire, we could not examine test–retest reliability, predictive validity or responsiveness to change over time; longitudinal research is needed. Third, causal relationships among social activities, *ikigai* and subjective health status should be investigated in a longitudinal survey.

## CONFLICT OF INTEREST

No conflict of interest has been declared by the authors.

## AUTHOR CONTRIBUTIONS

Hirano M: Study concept and design, acquisition of patients and/or data, and preparation of manuscript. Hirano M, Saeki K and Ueda I:Analysis and interpretation of data.

## ETHICAL APPROVAL

This study was performed after approval by the Ethics Committee of Hokkaido University Faculty of Health Sciences (approval no. 17–74). Participants’ provided informed oral consent and their anonymity were preserved. This study conforms to the provisions of the Declaration of Helsinki (as revised in Brazil 2013).
